# Gene expression analysis in subcutaneous adipose tissue reveals a predominant influence of lncRNAs during growth

**DOI:** 10.1016/j.gendis.2024.101351

**Published:** 2024-06-14

**Authors:** Federica Rey, Letizia Messa, Clarissa Berardo, Alessia Mauri, Gianvincenzo Zuccotti, Cristina Cereda, Stephana Carelli

**Affiliations:** a Pediatric Clinical Research Center "Romeo ed Enrica Invernizzi", Department of Biomedical and Clinical Science, University of Milan, Milan 20157, Italy; b Center of Functional Genomics and Rare Diseases, Department of Pediatrics, Buzzi Children's Hospital, Milan 20154, Italy; c Department of Electronics, Information and Bioengineering (DEIB), Politecnico di Milano, Milan 20133, Italy; d Department of Pediatrics, Buzzi Children's Hospital, Milan 20154, Italy

The adipose tissue is a crucial energy reservoir that can undergo significant changes during aging, impacting the pathogenesis of metabolic disorders, including obesity.^1^ Obesity affects individuals of all ages, with different implications for each stage of life. It is thought to be a state of accelerated aging, prompting the introduction of the term "adipaging", for which obesity and aging share key biological hallmarks strictly related to a dysfunctional adipose tissue.^2^ Understanding the complex molecular relationships between obesity and aging is crucial for developing effective strategies to mitigate their impact.^2^ As transcriptional studies can allow the identification of novel gene expression patterns and identify putative genes and regulatory pathways that could contribute to the development of diseases related to obesity, we conducted a computational integration of available total RNA sequencing datasets in obesity-affected patients, to obtain insights into the transcriptional differences present in the subcutaneous adipose tissue of obesity-affected children and adults in a wide cohort.

The Gene Expression Omnibus (GEO) Dataset repository was exploited to search for publicly available total RNA sequencing datasets pertaining to studies conducted in subcutaneous adipose tissue of obesity-affected adult and pediatric patients. Filtering criteria reported in Figure S1A yielded 8 publicly datasets for a total of 129 samples in obese adult patients (OBAD) and 2 datasets for a total of 34 samples in obese pediatric patients (OBPED). Information regarding tissue, gender, age, anthropometric features, and the number of samples were retrieved (Fig. S1A and Table S1). Datasets were re-processed and analysis on both coding and non-coding genes were performed (Fig. S1B) to explore obesity-related pathways and identify new putative regulatory pathways associated with its development. This computational approach enhances our understanding of obesity's molecular landscape highlighting new potential targets that could be further explored in future studies.

Principal component analysis of samples highlighted that OBAD and OBPED were separated and that the two groups presented a sub-division, suggesting potential influences beyond age, such as gender and ethnicity, on gene expression patterns (Fig. 1A). A volcano plot was built to highlight the dysregulation profile between OBAD versus OBPED (Fig. S2A). Genes showing |log_2_ fold change| ≥ 1 and a false discovery rate ≤0.1 were considered as differentially expressed (DE-RNAs) and retained for further analysis. A total of 3330 DE-RNAs were identified (Table S2 and Fig. 1B), and when we assigned the gene biotype, we found DE-RNAs to be predominantly non-coding genes (3140 out of 3330), with the dysregulation primarily driven towards up-regulation (2665 out of 3330). The 3'UTR length of DEGs was differentially distributed from that of the genome, with a tendency towards longer and more variable 3'UTR lengths (Fig. 1C). This is relevant, as it aligns with the role of lncRNA in modulating mRNA stability via binding to 3'UTRs.^3^ The DE-RNA distribution by gene type, as performed with ShinyGo prediction which identifies the expected distribution according to genome size, showed an interesting pattern with coding genes being significantly less than expected as opposed to ncRNAs, which showed an opposite pattern, suggesting the impact of non-coding RNAs in obesity (Fig. 1D). The coding DE-RNAs in OBAD versus OBPED were subjected to functional enrichment analysis using ShinyGO, highlighting deregulation in sensory perception and signal transduction (Fig. S2B). DE-RNA localization in the genome was evaluated, highlighting 17 enriched chromosomal regions (Fig. S2C).

**Figure 1:**
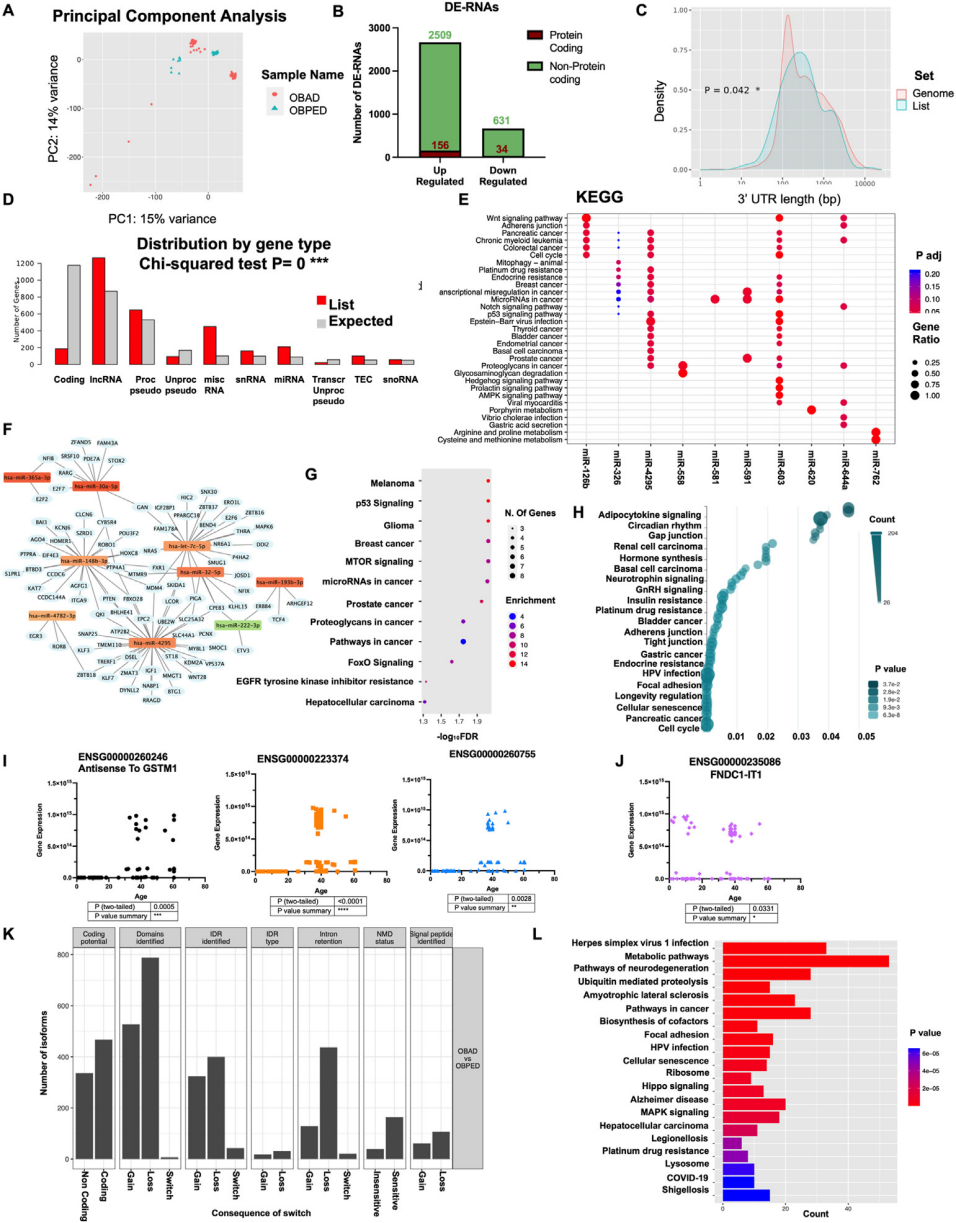
RNA dysregulation in adipose tissue during growth. **(A)** Principal component analysis of differentially expressed genes (DEGs) in OBAD and OBPED. Genes showing |log_2_ fold change| ≥ 1 and a false discovery rate (FDR) ≤ 0.1 were considered differentially expressed. **(D)** Differential expression analysis highlighted 3330 deregulated genes. **(C)** The density of 3'UTR length of DEGs compared with one of the genomes, with transcripts presenting a typically longer, though more variable, 3'UTR length. **(D)** The histogram representing the distribution of DEGs by gene type. **(E)** The dotplot representing the miRNA pathway analysis performed on KEGG. The *y*-axis represents the name of the pathways, the *x*-axis reports the miRNA family with the number of recognized targets in round brackets (*i.e.*, target with at least one annotation), the colors of the dots represent the adjusted *P*-values, and the size of the dots represents gene ratio (*i.e.*, the number of miRNA targets found annotated in each category over the total number of recognized targets indicated in round brackets). **(F)** Network analysis performed on Cytoscape between miRNAs and interacting genes. **(G)** Functional enrichment analysis of interacting genes. The *y*-axis represents the name of the pathway, the *x*-axis represents the FDR in logarithmic scale, the dot size represents the number of different DEGs in the pathway, and the color indicates the fold enrichment. **(H)** KEGG enrichment analysis of coding RNAs, miRNAs, and lncRNAs performed on ncPath. **(I)** Correlation analysis of age with the top 3 most significant up-regulated lncRNAs. **(J)** Correlation analysis of age with the top significant down-regulated lncRNA. **(K)** Overview of the number of switched isoforms predicted to have functional consequences. **(K)** KEGG functional enrichment analysis on switching genes involved in differential isoform expression. The *y*-axis represents the name of the pathway, the *x*-axis represents the number of genes involved in the pathway, and the color indicates the fold enrichment. OBAD, obese adult patients; OBPED, obese pediatric patients.

GSEA analysis for KEGG highlighted a positive enrichment in metabolic pathways and cancer-related pathways along with a negative enrichment in the TNF signaling pathway and insulin secretion (Fig. S3A). Moreover, analysis of the longevity regulating pathway indicated a decrease in genes associated with stress response and stem cell renewal in adults, suggesting an "aged" adipose tissue less responsive to stress stimuli (Fig. S3B). Gene ontology enrichment analysis highlighted the regulation of anion transport, transcriptional, and post-transcriptional processes as the most dysregulated biological processes (Fig. S3C). Moreover, among the most enriched cellular components appeared to be the Golgi, RNA inhibitor complexes, and chromosomal centromeric regions (Fig. S3D). GO molecular function also highlighted an implication for transcriptional and post-transcriptional processes (Fig. S3E). Investigation of diseases associated with dysregulated coding gene signatures revealed potential comorbidities such as heart, kidney, and inflammatory disorders (Fig. S3F).

Differential expression analysis highlighted 210 dysregulated miRNAs, of which 206 were up-regulated and 4 down-regulated (Table S2). Functional enrichment analysis using miRTarBase for KEGG (Fig. 1E), WikiPathways (Fig. S4A), Disease Ontology (Fig. S4B), and Reactome (Fig. S4C) highlighted enrichment of miRNAs in cancer-related processes, suggesting varying cancer susceptibility between adult and pediatric obesity-affected patients. Moreover, the network analysis allowed the identification of possible miRNAs interacting genes (Fig. 1F), and enrichment analysis of these interacting genes highlighted numerous cancer-related pathways as dysregulated (Fig. 1G). We also identified 1314 dysregulated lncRNAs in our dataset (Table S2), and we decided to perform a joint KEGG enrichment analysis of coding RNA, miRNAs, and lncRNAs (Fig. 1H; Fig. S4D). This analysis uncovered dysregulation in pathways such as the adipocytokine signaling pathway and the longevity regulating pathway, suggesting a potential role for specific lncRNAs in these processes. Additionally, we investigated the expression patterns of the top 3 most significantly up-regulated (Fig. 1I) and down-regulated (Fig. 1J; Fig. S4E) lncRNAs across different ages, highlighting a strong significance for up-regulated transcripts in older subjects, supporting the potential involvement of these lncRNAs in age-related mechanisms.

We analyzed variations in splicing events and isoform levels, identifying 2860 differentially expressed isoforms in OBAD patients with switching features associated with 1444 unique genes, mostly protein-coding genes (Table S3 and Fig. S5A). Alternative transcription start site and alternative transcription termination site events were the most predominant, with 7580 and 7355 events respectively, followed by Exon skipping (5866 events), alternative 5' donor site (5591 events), alternative 3' acceptor site (4990 events), multi-exon skipping (2687 events), intron retention (2168 events), and mutually exclusive exons with only 66 events (Fig. S5B, C). The most frequent changes observed affected protein domains, coding potential, disordered regions, and intron retention (Fig. 1K; Fig. S5D). Given the evidence from alternative splicing and switching isoform analysis, we performed a KEGG functional enrichment analysis on all switching genes identified, highlighting aging-related diseases such as neurodegenerative disorders, cellular senescence, metabolic pathways, and cancer pathways (Table S4 and Fig. 1L).

In conclusion, we identified distinct gene expression patterns and dysregulated pathways in childhood and adult obesity using publicly available RNA sequencing datasets. Specifically, it is of key relevance that most of the dysregulated genes are non-coding RNAs, and this gives rise to several new targets worth investigating and characterizing.^4^ Although speculative, we could hypothesize that epigenetic marks accumulate during aging thus favoring the differential expression of regulatory mechanisms such as lncRNA and isoform switching, which ultimately impact gene expression. This underscores the importance of exploring non-coding RNAs in future *in vitro* and *in vivo* studies, as they could be novel targets for in adipaging. Moreover, the discovery of these novel non-coding RNAs holds the potential to illuminate previously unknown mechanisms underlying obesity pathogenesis, potentially serving as a bridge between lifestyle factors and disease development. By shedding light on the role of non-coding RNAs in obesity, our study paves the way for future studies to validate these dysfunctions. Through our comprehensive analysis, we provide valuable insights that can inform future research directions and ultimately lead to improved management strategies for individuals affected by obesity.

## Author contributions

F.R., L.M.: conceptualization, work design, data acquisition and interpretation, and writing and revising of the manuscript; C.B., A.M.: data acquisition and interpretation; G.Z.: funding acquisition and supervision; C.C.: data interpretation, funding acquisition, manuscript revision, and supervision; S.C.: conceptualization, work design, data interpretation, writing and revising of the manuscript, supervision, and funding acquisition.

## Conflict of interests

The authors declared no conflict of interests.

## Figures and Tables

**Figure S1:**
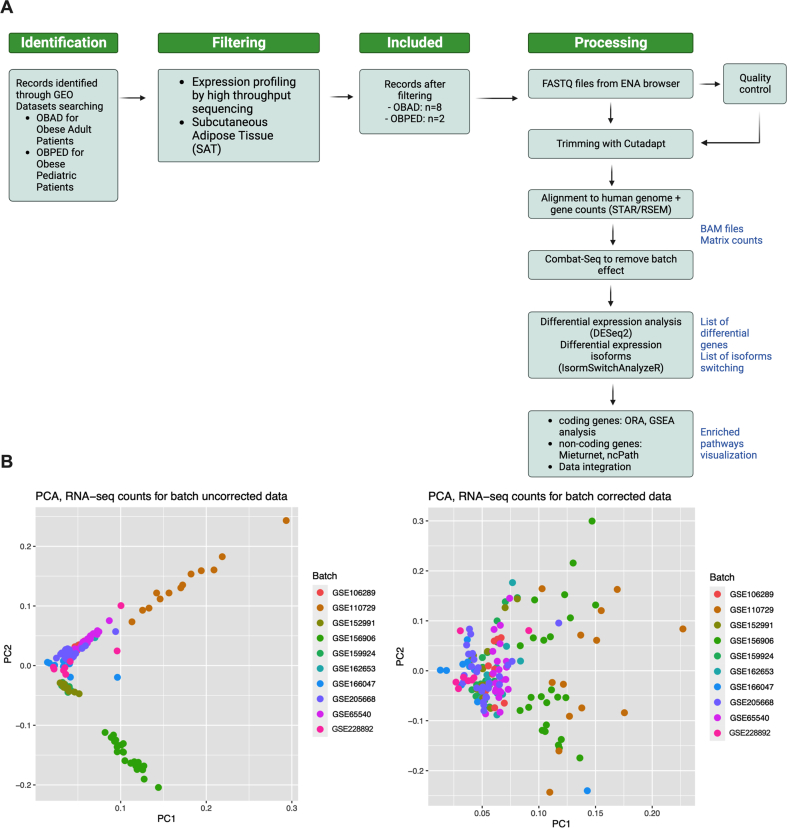
RNA sequencing datasets retrieval and analysis workflow. **(A)** GEO repository was considered for RNA sequencing datasets related to subcutaneous adipose tissue of human subjects. Raw FASTQ files were processed with a common and standardized pipeline to remove any potential batch effects. **(B)** Principal component analysis of samples before and after batch effect removal via Combat-Seq function.

**Figure S2:**
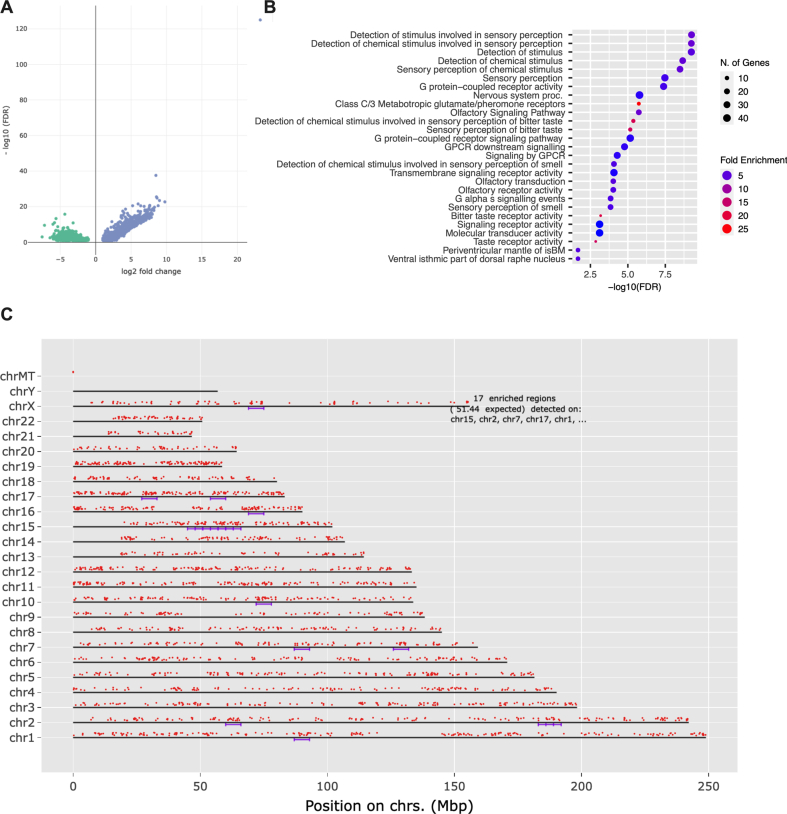
Global transcriptional deregulation in coding and non-coding genes is present in subcutaneous adipose tissue from OBAD and OBPED. **(A)** The volcano plot showing deregulated genes between adult and pediatric obese patients. On the *x*-axis is reported the log_2_ fold change whereas on the *y*-axis is reported the false discovery rate (FDR) in logarithmic scale. **(B)** Functional enrichment analysis of coding deregulated genes in subcutaneous adipose tissue from OBAD and OBPED. The *y*-axis represents the name of the pathway, the *x*-axis represents the FDR in logarithmic scale, the dot size represents the number of different differentially expressed genes (DEGs) in the pathway, and the color indicates the fold enrichment. **(C)** DEG localization in the genome. Enriched regions are highlighted with a purple line. OBAD, obese adult patients; OBPED, obese pediatric patients.

**Figure S3:**
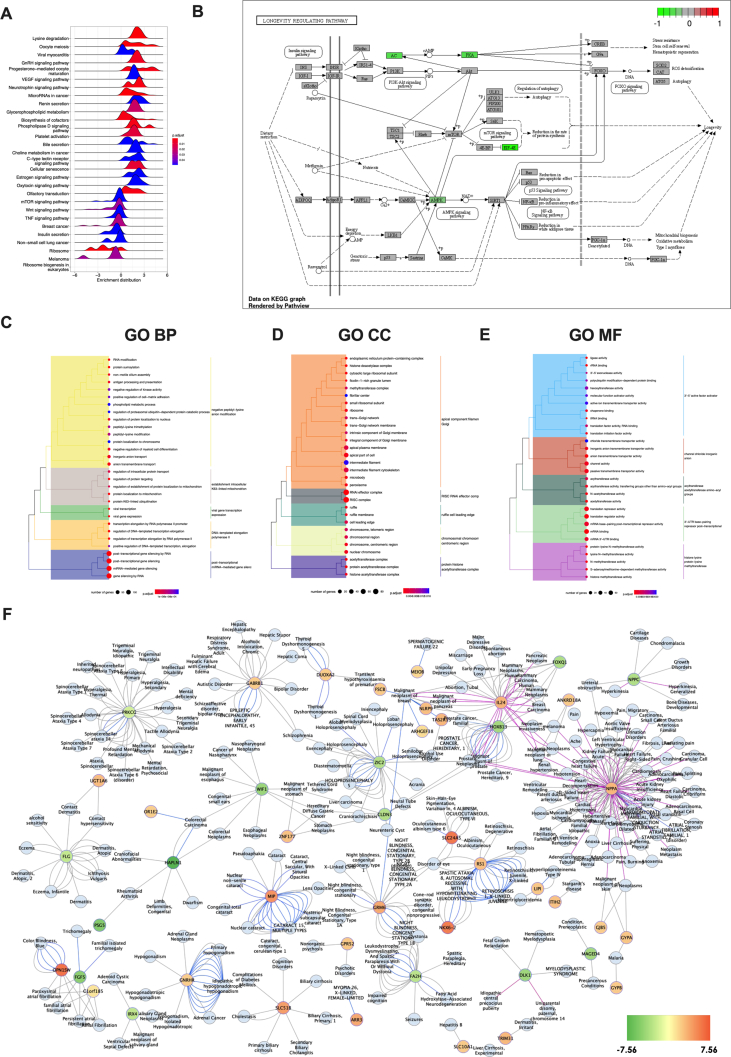
Functional enrichment analysis of adult and pediatric adipose tissues. **(A)** Ridgeplot visualization of core enriched genes for GSEA. KEGG pathway analysis highlighted a positive enrichment in metabolic pathways and cancer-related pathways whilst it showed an interesting negative enrichment in the TNF signaling pathway and insulin secretion. **(B)** Pathview of KEGG "longevity regulating pathway" highlighting the up-regulated differentially expressed genes (DEGs) in green. **(C–E)** The tree plot showing the hierarchical clustering of enriched terms in GO BP, GO CC, and GO MF, respectively. Pathways are clustered according to pairwise similarities which are calculated according to Jaccardi's similarity index; the dot size represents the number of different DEGs in the pathway, and the color indicates the adjusted *P*-value. **(F)** DisGeNET analysis shows the terms implicated in obesity in subcutaneous adipose tissue from OBAD and OBPED. The lines connecting the genes to the disease term represent the evidence reported in the literature for the terms' implication in the disease. The color scale for the genes represents the gene fold change. OBAD, obese adult patients; OBPED, obese pediatric patients; GO, gene ontology; BP, biological process; MF, molecular function; CC, cellular component.

**Figure S4:**
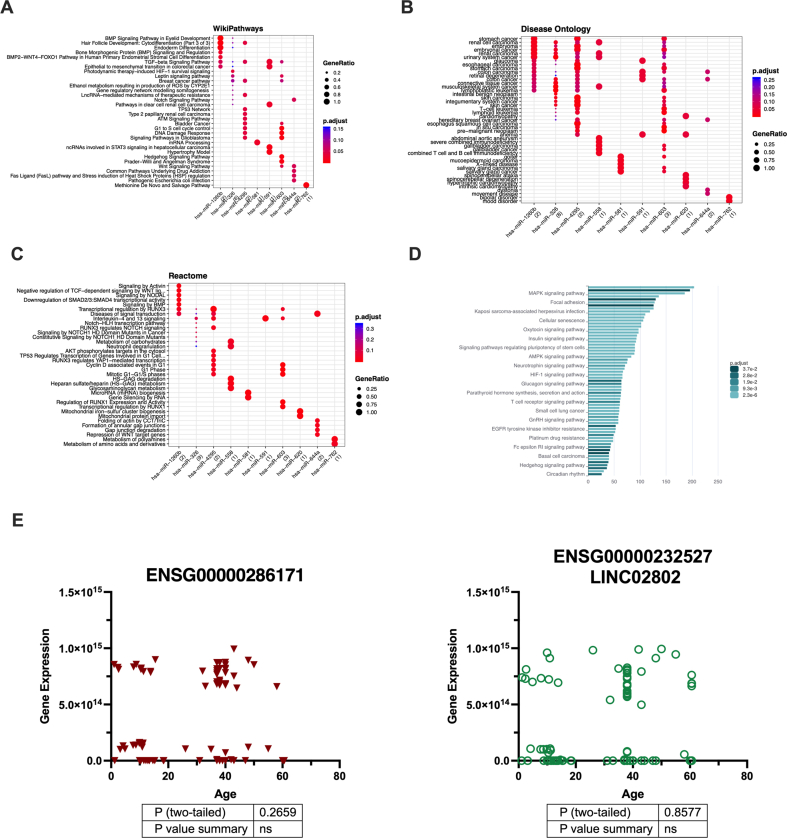
Influence of aging in non-coding expression profiles. **(A–C)** The dotplot representing the miRNA pathway analysis performed on WikiPathways, Disease Ontology, and Reactome, respectively. The *y*-axis represents the name of the pathways, the *x*-axis reports the miRNA family with the number of recognized targets in round brackets (*i.e.*, target with at least one annotation), the colors of the dots represent the adjusted *P*-values, and the size of the dots represents gene ratio (*i.e.*, the number of miRNA targets found annotated in each category over the total number of recognized targets indicated in round brackets). **(D)** KEGG enrichment analysis of coding RNAs, miRNAs, and lncRNAs performed on ncPath. **(E)** Correlation analysis of age with the other top 2 most significant down-regulated lncRNAs.

**Figure S5:**
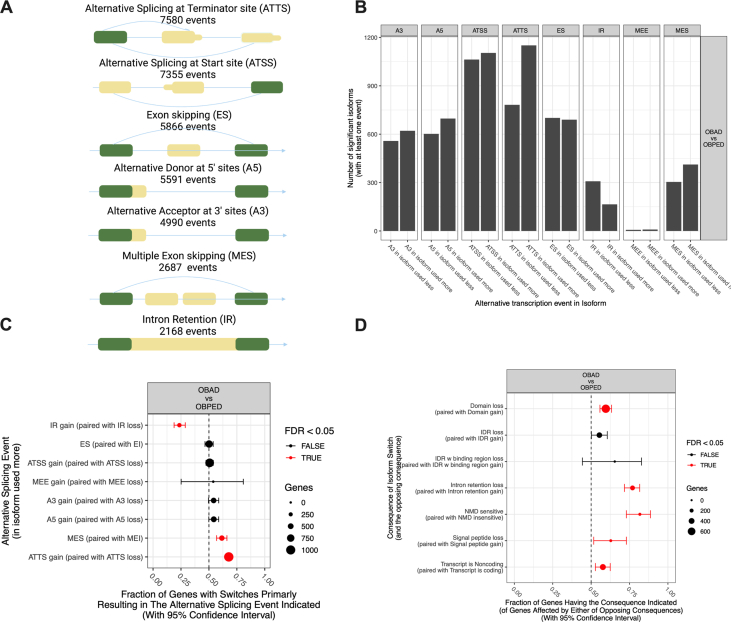
Genome-wide transcripts analysis for switched isoforms between OBAD and OBPED. **(A)** Illustration of alternative splicing event types for the switched isoforms. Created with BioRender.com. **(B, C)** Distribution of isoform usage (increased or decreased dIF) in each type of alternative splicing. **(D)** Overview of the number of switched isoforms predicted to have functional consequences. OBAD, obese adult patients; OBPED, obese pediatric patients.

## Appendix A Supplementary data

Multimedia component 1 Multimedia component 1

Table S1 Information regarding all the datasets considered such as tissue, gender, age, anthropometric features, and the number of samples. Table S1

Table S2 List of differentially expressed genes in OBAD versus OBPED samples calculated via DESeq2. Note: For each gene, the mean of normalized counts for all samples (baseMean), the log_2_ fold change, the standard error (*i.e.*, lfcSE), the Wald statistic (*i.e.*, stat), the Wald test *P*-value, the false discovery rate adjusted *P*-value, the gene name, and gene type (lncRNA or protein-coding) are reported. OBAD, obese adult patients; OBPED, obese pediatric patients. Table S2

Table S3 List of differentially expressed isoforms in OBAD versus OBPED samples calculated via IsoformSwitchAnalyzeR. Note: Transcript-specific information includes gene identification (gene_id) and gene name (gene_name) alongside the considered conditions (condition_1: OBAD and condition_2: OBPED). The isoform fraction (IF), representing the ratio of isoform expression to overall gene expression, is provided for each condition. The difference in isoform usage (dIF), calculated as IF2-IF1, and associated q-values are reported. Additionally, instances where the dIF denotes a consequential switch in isoform usage within the gene are highlighted. OBAD, obese adult patients; OBPED, obese pediatric patients. Table S3

Table S4 List of pathways associated with genes undergoing isoform switch consequences obtained via g:Profiler. Note: This table enumerates KEGG pathways linked to genes exhibiting a switch consequence in isoform usage. For each pathway, the KEGG-specific ID, the adjusted *P*-value, the number of genes of the pathway (term size), and the number of genes with a switch in the specific pathway are reported. Table S4
