# Supplementary material for: Gene expression analysis in subcutaneous adipose tissue reveals a predominant influence of lncRNAs during growth
Source: Genes Dis. 2024 Jun 14;12(2):101351. doi: 10.1016/j.gendis.2024.101351 (PMC11625322; doi:10.1016/j.gendis.2024.101351)
Supplement: Multimedia component 1 [file mmc1.docx]

**Materials & Methods**

**RNA-Seq dataset selection**

The Gene Expression Omnibus (GEO) Dataset repository was exploited to search for publicly available RNA-Seq datasets pertaining studies conducted in Subcutaneous Adipose Tissue (SAT) of obesity-affected adult and pediatric patients. By considering “Homo Sapiens” as organism, “Obesity” as search term and “High-throughput sequencing” as study type, a total of 211 publicly available datasets related to obesity were obtained. Filtering for studies performed on SAT, a total of 10 publicly, available obesity-affected adult and pediatric patients’ datasets were considered (Fig. S1A). For each dataset, information regarding tissue, gender, age, anthropometric features and the number of samples were retrieved (Table S1).

**Data Reprocessing and Differential Expression Analysis**

As the samples selected came from different sources and they were previously processed with different libraries, protocols, and tools, the raw FASTQ files were downloaded from the European Nucleotide Archive (ENA) browser and reprocessed using a pipeline common to all the datasets to obtain comparable data. First, a per-base quality check of the sequenced reads was performed on FastQC, a quality control tool for high throughput sequence data. Then, where needed according to the FastQC report, the adapter sequences were removed using the Cutadapt software. The reads were mapped against the human reference genome with STAR with Gencode GRCh38.p13 release and quantified with RSEM to assess transcript abundances and generate the read count matrix. To avoid any possible remaining biases in data the ComBat-Seq R package was used ^1^. Specifically, this is a tool for batch effect adjustment for RNA-Seq count data which exploits a negative binomial regression to model batch effects and provide adjusted data by mapping the original data to an expected distribution if there were no batch effects. To perform the differential expression and pathways analysis the raw count data were analyzed on iDEP (integrated Differential Expression and Pathway analysis; <http://ge-lab.org/idep/>) ^2^. iDEP tool was used to obtain Principal Component Analysis (PCA) plot, Heatmap plot, Volcano Plot and Pathview plots. The differential expression analysis was performed on R with the DESeq2 packages by considering |log_2_FC|≤ 1 and FDR < 0.1. Pathway analysis for Differentially Expressed Genes (DEGs) was performed using ShinyGO ^3^. ShinyGo was also used to obtain information on distribution by gene type, 3’ UTR length and genome localization of coding DEGs. GSEA (Gene Set Enrichment Analysis) was used to assess global perturbations in gene expression and enrichment analyses were performed for KEGG, Gene Ontology Biological Processes (GO BP), Gene Ontology Cellular Component (GO CC), Gene Ontology Molecular Function (GO MF) and visualized as treeplot ^4^. The GSEA of selected pathways was displayed with pathview ^5^. DisGeNET tool on Cytoscape was exploited to assess coding deregulated genes implications in obesity ^6^ (Fig S1).

**Non-Coding RNAs Bioinformatics Analyses**

The differentially expressed miRNAs were analyzed for their functional enrichment with the Mienturnet webtool ^7^ performing a miRNA-target Enrichment analysis with miRTarBase for KEGG, Reactome, WikiPathways and Disease ontology and performing a network analysis visualized via Cytoscape ^8^. The mRNAs interacting with the miRNAs identified through this network were loaded on the ShinyGo webtool for enrichment analysis with KEGG and Reactome ^3^. The differentially expressed miRNAs, lncRNAs and coding genes were loaded on the ncpath webtool and an integrated KEGG enrichment analysis which includes all three classes of RNAs was performed ^9^.

**Analysis of Alternative Splicing Isoforms and Functional Consequences**

The statistical identification of isoform switching in OBAD samples with respect to OBPED samples isoforms was performed via IsoformSwitchAnalyzeR R package ^10^. Isoforms’ count table generated by RSEM were imported via the ImportRdata() function ^11^. Then, the DRIMSeq R package was used to identify isoform switches in the comparison considered ^12,13^. Specifically, isoforms were considered differentially switched and retained for further analysis with difference in isoform fraction (dIF) > 0.1 and FDR < 0.05. The functional consequences of switched isoforms were first analyzed for Nonsense-mediated decay (NMD) status, open reading frames (ORF), and intron retention. Moreover, other biological implications such as protein-coding potential, protein domains, intrinsically disordered regions (IDR), and signal peptide were predicted via external tools ^14–17^. The alternative splicing (AS) patterns of switching isoforms were predicted by spliceR to include alternative 3′ acceptor sites (A3), alternative 5′ donor sites (A5), exon skipping (ES), mutually exclusive exons (MEE), AS at TF start sites (ATSS), AS at termination site (ATTS), and intron retention (IR) ^10,12,18^.

**Isoforms pathways analysis**

Functional enrichment analysis was performed via g:Profiler web tool (https://biit.cs.ut.ee/gprofiler/gost; ^19^), dividing isoforms according to the specific isoform switch they faced (e.g. coding potential, protein domains, NMD status, IDR, intron retention and signal peptide) and using Bonferroni-Hochberg FDR correction of 0.05 as threshold. Kyoto Encyclopedia of Genes and Genomes (KEGG) (<https://www.genome.jp/kegg/>; ^20^) of specific differentially switched isoforms was performed. The R software was then used to generate the dotplot graphs (ggplot2 R package; ^21^).

**References**

1. Zhang Y, Parmigiani G, Johnson WE. ComBat-seq: batch effect adjustment for RNA-seq count data. *NAR Genomics and Bioinformatics*. 2020;2(3):lqaa078. doi:10.1093/nargab/lqaa078

2. Ge SX, Son EW, Yao R. iDEP: an integrated web application for differential expression and pathway analysis of RNA-Seq data. *BMC Bioinformatics*. 2018;19(1):534. doi:10.1186/s12859-018-2486-6

3. Ge SX, Jung D, Yao R. ShinyGO: a graphical gene-set enrichment tool for animals and plants. Valencia A, ed. *Bioinformatics*. 2020;36(8):2628-2629. doi:10.1093/bioinformatics/btz931

4. Wu T, Hu E, Xu S, et al. clusterProfiler 4.0: A universal enrichment tool for interpreting omics data. *The Innovation*. 2021;2(3):100141. doi:10.1016/j.xinn.2021.100141

5. Luo W, Brouwer C. Pathview: an R/Bioconductor package for pathway-based data integration and visualization. *Bioinformatics*. 2013;29(14):1830-1831. doi:10.1093/bioinformatics/btt285

6. Piñero J, Bravo À, Queralt-Rosinach N, et al. DisGeNET: a comprehensive platform integrating information on human disease-associated genes and variants. *Nucleic Acids Res*. 2017;45(D1):D833-D839. doi:10.1093/nar/gkw943

7. Licursi V, Conte F, Fiscon G, Paci P. MIENTURNET: an interactive web tool for microRNA-target enrichment and network-based analysis. *BMC Bioinformatics*. 2019;20(1):545. doi:10.1186/s12859-019-3105-x

8. Shannon P, Markiel A, Ozier O, et al. Cytoscape: a software environment for integrated models of biomolecular interaction networks. *Genome Res*. 2003;13(11):2498-2504. doi:10.1101/gr.1239303

9. Li Z, Zhang Y, Fang J, et al. NcPath: a novel platform for visualization and enrichment analysis of human non-coding RNA and KEGG signaling pathways. Kendziorski C, ed. *Bioinformatics*. 2023;39(1):btac812. doi:10.1093/bioinformatics/btac812

10. Vitting-Seerup K, Sandelin A. IsoformSwitchAnalyzeR: analysis of changes in genome-wide patterns of alternative splicing and its functional consequences. *Bioinformatics*. 2019;35(21):4469-4471. doi:10.1093/bioinformatics/btz247

11. Soneson C, Love MI, Robinson MD. Differential analyses for RNA-seq: transcript-level estimates improve gene-level inferences. *F1000Res*. 2015;4:1521. doi:10.12688/f1000research.7563.2

12. Vitting-Seerup K, Sandelin A. The Landscape of Isoform Switches in Human Cancers. *Mol Cancer Res*. 2017;15(9):1206-1220. doi:10.1158/1541-7786.MCR-16-0459

13. Nowicka M, Robinson MD. DRIMSeq: a Dirichlet-multinomial framework for multivariate count outcomes in genomics. *F1000Res*. 2016;5:1356. doi:10.12688/f1000research.8900.2

14. Ritchie ME, Phipson B, Wu D, et al. limma powers differential expression analyses for RNA-sequencing and microarray studies. *Nucleic Acids Research*. 2015;43(7):e47-e47. doi:10.1093/nar/gkv007

15. Punta M, Coggill PC, Eberhardt RY, et al. The Pfam protein families database. *Nucleic Acids Research*. 2012;40(D1):D290-D301. doi:10.1093/nar/gkr1065

16. Almagro Armenteros JJ, Tsirigos KD, Sønderby CK, et al. SignalP 5.0 improves signal peptide predictions using deep neural networks. *Nat Biotechnol*. 2019;37(4):420-423. doi:10.1038/s41587-019-0036-z

17. Mészáros B, Erdos G, Dosztányi Z. IUPred2A: context-dependent prediction of protein disorder as a function of redox state and protein binding. *Nucleic Acids Res*. 2018;46(W1):W329-W337. doi:10.1093/nar/gky384

18. Vitting-Seerup K, Porse BT, Sandelin A, Waage J. spliceR: an R package for classification of alternative splicing and prediction of coding potential from RNA-seq data. *BMC Bioinformatics*. 2014;15:81. doi:10.1186/1471-2105-15-81

19. Raudvere U, Kolberg L, Kuzmin I, et al. g:Profiler: a web server for functional enrichment analysis and conversions of gene lists (2019 update). *Nucleic Acids Res*. 2019;47(W1):W191-W198. doi:10.1093/nar/gkz369

20. Kanehisa M, Goto S. KEGG: kyoto encyclopedia of genes and genomes. *Nucleic Acids Res*. 2000;28(1):27-30. doi:10.1093/nar/28.1.27

21. Wickham H. *Ggplot2: Elegant Graphics for Data Analysis*. Springer; 2009.
