# Supplementary material for: Gene expression analysis in subcutaneous adipose tissue reveals a predominant influence of lncRNAs during growth
Source: Genes Dis. 2024 Jun 14;12(2):101351. doi: 10.1016/j.gendis.2024.101351 (PMC11625322; doi:10.1016/j.gendis.2024.101351)
Supplement: Table S1 — Information regarding all the datasets considered such as tissue, gender, age, anthropometric features, and the number of samples. [file mmc2.pdf]

Supplementary Table S1. Information regarding all the datasets considered such as tissue, gender, age, anthropometric features and the number of samples

	ADULTS OBESE									PEDIATRIC OBESE	
	GSE159924	GSE156906	GSE152991	GSE162653	GSE110729	GSE106289	GSE65540	GSE166047		GSE205668	GSE228892
# Sample	21	28	15	10	13	10	22	10	# Sample	26	8
Tissue	SAT	SAT	SAT	SAT	SAT	SAT	SAT	SAT	Tissue	SAT	SAT
Gender	Female and male	/	Female and male	Female and male	Female	Female	Female	Female and male	Gender	Female and Male	Male
Age (y)	40 ± 1	38 ±1	37 ± 2	44.55 ± 12.14	39 ± 7	60.6 ± 3.6	37.5 ± 8.3	43.2 ± 8.5	Age	/	9.54 ± 1.6
Weight (kg)	106.0 ± 3.6	/	/	/	51 ± 4	101.3 ± 9.7	/	112 ± 13.4	Weight (kg)	/	54.96 ± 12.4
BMI (kg/m2)	37.8 ± 1.1	37.7 ± 0.9	36.9 ± 1.3	34.80 ± 2.87	/	38.8 ± 3.4	46.5 ± 5.6	38 ± 4.4	Height (cm)	/	137.53 ± 10.5
Body fat (%)	47.7 ± 1.4	48.0 ± 1.1	47.2 ± 1.9	41.52 ± 6.84	/	51.4 ± 6.1	/	/	Body mass index (BMI)		
SAAT (cm3)	3.705 ± 236	3701 ± 181	3624 ± 292	/	/	/	/	/	-kg/m2	/	26.375 ± 1.1
IAAT volume (cm3)	1.073 ± 97	938 ± 89	1014 ± 129	/	/	/	/	/	-z-score	/	2.835 ± 0.4
IHTG content (%)	2.6 ± 0.2	2.4 ± 0.2	2.3 ± 0.2	/	/	/	/	/	Fasting blood glycemia (mg/dL; nv<100 mg/dl)	/	99.665 ± 2.3
Fasting insulin (µU/mL)	10.7 ± 0.8	12.3 ± 1.3	13 ± 2	/	/	12.3 ± 1.8	21.6 ± 10.9	/	Insulin	/	19.015 ± 4.97
HOMA-IR	/	/	2.9 ± 0.5	1.64 ± 0.79	3.8 ± 1.6	3.30 ± 0.59	/	/	Triglycerides (mg/dL)	/	

Fasting glucose (mg/dL)	87.8 ± 0.9	88 ±1	88 ± 1	100.8 ± 18.9	95.4 ± 10.8	103.4 ± 17.6	/	/	(nv ≥ 130 mg/dL if ≥ 10 years)	/	124.415 ± 14.4
OGTT 2-h glucose (mg/dL)	105.9 ± 3.3	107 ± 3	107 ± 4	/	/	/	/	/	HDL-cholesterol (mg/dL) (nv > 50 in males)	/	51.415 ± 4.2
HbA1c (%)	5.0 ± 0.1	5.1 ± 0.1	5.1 ± 0.1	/	/	/	/	/	Triglycerides/ HDL-cholesterol ratio (nv < 2.2)	/	2.45 ± 0.43
Triglycerides (mg/dL)	71 ± 5	69 ± 4	67 ± 6	23.8 ± 12.6	21.6 ± 10.8	96.9 ± 28.8	23.4 ± 10.6	176.4 ± 71.8	Triglyceride–glucose index (nv < 7.88)	/	8.72 ± 0.15
HDL-cholesterol (mg/dL)	55 ± 3	/	50 ± 3	25.6 ± 6.7	23.4 ± 5.4	54.3 ± 10.3	20 ± 5.8	40 ± 9.9	HOMA-IR	/	4.745 ± 1.26
LDL-cholesterol (mg/dL)	101 ± 6	/	99 ± 8	62.8 ± 14	79.2 ± 12.6	131.2 ± 24.8	56.5 ± 10.4	112 ± 20.6			
ALT (U/L)	17 ± 2	16 ±1	/	/	/	/	/	42 ± 46.7			
AST (U/L)	18 ± 1	17 ±1	/	/	/	/	/	23.3 ± 17			
Alkaline Phosphatase (U/L)	67 ± 4	/	/	/	/	/	/	/			
Bilirubin (mg/dL)	0.38 ± 0.03	/	/	/	/	/	/	/			
Hepatic insulin sensitivity index [1.000/(µmol/kgFFM/min × µU/mL)]	5.6 ± 0.3	5.6 ± 0.4	5.4 ± 0.4	/	/	/	/	/			
Glucose disposal rate during the clamp (µmol/kgFFM/min)	48.6 ± 2.8	383 ± 33	362 ± 35	/	/	/	/	/			

	Mean ± SEM			Mean ± SD							
